# Treatment of Chronic Catarrhal Ophthalmia

**Published:** 1844-12

**Authors:** 


*Treatment of Chronic Catarrhal Ophthalmia.*—M. Reveille-Parise strongly recommends the decoction, or a strong infusion of rhatany root, and a lotion with which the affected eyes are to be bathed. Besids acting as an astringent, this remedy seems to have some other mode of operation; for we do not find that similar preparations of oak-bark or of gall-nuts—although bath of these contain a large proportion of tannin—are equally efficacious, as collyria in the ophthalmia alluded to. The application should be used luke-warm, and a few drops Goulard's Extract may be added to it, if deemed proper.—*Ibid.*

